# Urban–rural prostate cancer disparities in a regional state of Australia

**DOI:** 10.1038/s41598-022-06958-2

**Published:** 2022-02-22

**Authors:** Georgea R. Foley, C. Leigh Blizzard, Brian Stokes, Marketa Skala, Frank Redwig, Joanne L. Dickinson, Liesel M. FitzGerald

**Affiliations:** 1 Menzies Institute for Medical Research, University of Tasmania, TAS, 17 Liverpool St, Hobart, 7000 Australia; 2 WP Holman Clinic, Royal Hobart Hospital, Hobart, TAS Australia; 3 Urology Department, Royal Hobart Hospital, Hobart, TAS Australia

**Keywords:** Cancer epidemiology, Urological cancer

## Abstract

Men living in regional and remote areas experience disparities in prostate cancer (PrCa) diagnosis, clinical characteristics and treatment modalities. We sought to determine whether such disparities exist in PrCa patients from Tasmania; a regional state of Australia with the second-highest rate of diagnosis and where over a third of residents live in outer regional and remote areas. Our study included clinicopathological data from 1526 patients enrolled in the Prostate Cancer Outcomes Registry-Tasmania. Regression analyses were undertaken to determine whether demographic, clinical and treatment variables differed between inner regional and outer regional/remote patients. Men from outer regional/remote areas were significantly more likely to reside in lower socio-economic areas, be diagnosed at a later age and with more clinically aggressive features. However, in contrast to previous studies, there were no overall differences in diagnostic or treatment method, although men from outer regional/remote areas took longer to commence active treatment and travelled further to do so. This study is the first to investigate PrCa disparities in a wholly regional Australian state and highlights the need to develop systematic interventions at the patient and healthcare level to improve outcomes in outer regional and remote populations in Australia and across the globe.

## Introduction

Prostate cancer is the most common form of non-cutaneous carcinoma in Australian men^1^. Over the last decade, there has been a considerable increase in PrCa incidence throughout the country, with figures continuing to rise annually. Tasmanian health resources are particularly challenged by PrCa prevalence. Each year, around 500 cases, predominantly of Caucasian ancestry, are diagnosed within the state and approximately 75 individuals die of the disease. According to data from the Australian Institute for Health and Welfare (AIHW), Tasmanian age-standardised incidence (age-standardised to the Australian 2001 male population; 2010–2014; 146.0 per 100,000) and mortality (2012–2016; 28.1 per 100,000) rates are more similar to those seen in the whole of Australia outer regional (160.8 and 29.8 per 100,000, respectively) and remote (140.1 and 26.5 per 100,000, respectively) populations than in Australian major cities (162.2 and 24.2 per 100,000, respectively). Notably, the most urbanised areas of Tasmania are only classed as inner regional, not major cities, according to the Australian Bureau of Statistics (ABS) produced Accessibility and Remoteness Index of Australia, and over a third of the population live in outer regional and remote areas.

Persons in outer regional and remote areas of Australia access diagnostic and treatment services less often than their urban counterparts^2,3^. A study utilising data from the Movember-funded Prostate Cancer Outcomes Registry-Victoria (PCOR-VIC) observed that males residing in a particular rural region of the state were older at diagnosis and more likely to have disease detected during other health procedures, rather than through preventative testing or monitoring^4^. These individuals presented with significantly higher prostate-specific antigen (PSA) levels and Gleason scores at diagnosis and also experienced a prominent delay between diagnosis and the commencement of treatment^5^, a factor that could adversely affect outcomes^6^. Furthermore, a nationwide study in 2005 identified 29% lower rates of radical prostatectomy in outer regional and rural areas^3^, and though regular PSA testing is undertaken across Australia, it is more common in capital cities^3^ and less in regional and remote areas^7^. A New Zealand study has reported similar discrepancies between rates of PSA screening and men living in rural or urban regions^8^. These screening, diagnostic and clinical discrepancies could underlie the higher rates of PrCa-specific mortality that have been repeatedly reported in patients from Australian outer regional and remote areas when compared to those from urban areas^2,3,5^.

Given the distinct population demographics, where over a third of the population live in outer regional and remote areas, we sought to investigate whether disparities in PrCa diagnosis, clinical features and treatment modalities exist in Tasmania, similar to those observed across mainland Australia and internationally. To do this, we utilised the Prostate Cancer Outcomes Registry-Tasmania (PCOR-TAS), an ongoing, opt-out registry that collects clinical and quality-of-life data from Tasmanian men diagnosed with PrCa. Through analysis of these data, we aimed to provide insight into where and why these disparities occur to inform and target interventions where they are most needed, potentially leading to better outcomes in regional populations.

## Results

A total of 1526 individuals with PrCa were enrolled in PCOR-TAS between 01/02/2015 and 19/07/2019. Participant characteristics are presented in Table 1. Due to the small size of the Tasmanian population, the most densely populated urban regions of the state are classed as ‘inner regional’. Of the 1526 individuals enrolled in PCOR-TAS, 976 resided in an ‘inner regional’ location (64%), and 550 individuals (36%) resided in an ‘outer regional/remote’ location (comprised of ‘outer regional’, ‘remote’, or ‘very remote’ postcodes). The inner regional and outer regional/remote groups differed in the distribution of age and ABS-developed Socio-Economic Indexes for Areas (SEIFA) index, and in terms of clinical characteristics other than method of diagnosis.

**Table 1:** Demographic and clinical characteristics of men with prostate cancer in inner and outer regional/remote areas of Tasmania.

	Inner regional % (n*)	Outer regional/remote % (n*)	*p*
Total participants	64.0% (976)	36.0% (550)	
**Age at diagnosis**			**< 0.001**
< 60	17.5% (171)	12.4% (68)	
60–64	20.4% (199)	14.2% (78)	
65–69	22.3% (217)	28.1% (155)	
≥ 70	39.8% (389)	45.4% (249)	
Age at diagnosis Median (IQR)	68.15 (62.24–73.88)	69.28 (64.63–74.49)	**0.001**
**SEIFA quintile**			**< 0.001**
1 (Most disadvantaged)	14.2% (139)	13.5% (74)	
2	14.1% (138)	44.0% (242)	
3	8.9% (87)	22.5% (124)	
4	29.7% (289)	16.5% (91)	
5 (Most advantaged)	33.1% (323)	3.5% (19)	
**Method of diagnosis**			0.33
TRUS	26.2% (255)	22.3% (123)	
TURP	10.0% (98)	11.4% (62)	
TP biopsy	60.8% (593)	64.2% (353)	
Other	1.9% (18)	1.8% (10)	
Missing	1.2% (12)	n.a. (< 5)	
**Diagnostic PSA (ng/mL)**			**0.004**
≤ 4	10.5% (102)	6.6% (36)	
4.01–10	51.1% (499)	45.2% (248)	
10.01–20	18.6% (181)	24.1% (133)	
> 20.01	11.2% (109)	11.8% (65)	
Missing	8.8% (85)	12.3% (68)	
**Gleason score**			**0.005**
< 7	36.0% (351)	28.4% (156)	
7 (3 + 4)	25.8% (252)	25.7% (141)	
7 (4 + 3)	13.6% (133)	17.4% (95)	
≥ 8	21.9% (214)	26.6% (146)	
Missing	2.8% (27)	2.0% (11)	
**NCCN risk**			**< 0.001**
Low	27.8% (271)	19.5% (107)	
Intermediate	37.6% (367)	39.3% (216)	
High	23.8% (233)	31.0% (170)	
Very high/metastatic	10.3% (101)	10.3% (56)	
Missing	0.5% (5)	n.a. (< 5)	
**Diagnostic facility**			**< 0.001**
Public	23.5% (230)	37.0% (204)	
Private	74.3% (725)	60.5% (333)	
Missing	2.1% (21)	2.6% (14)	
**Treatment facility (active treatment)**			**0.01**
Public	26.0% (254)	33.2% (182)	
Private	28.1% (274)	25.1% (138)	
Missing	6.8% (66)	3.8% (21)	
NA	39.1% (382)	38.0% (209)	

IQR = Interquartile Range; SEIFA = Socio-Economic Index of Advantage and Disadvantage; TRUS = Transrectal Ultrasound; TURP = Transurethral Resection of the Prostate; TP Biopsy = Transperineal Biopsy; PSA = Prostate Specific Antigen; NCCN = National Comprehensive Cancer Network; n.a. = Not Applicable.

*All analyses are weighted for remoteness classification (see “Methods”). Subject numbers are rounded to the nearest integer value.

Table 2 compares the proportions of inner regional and outer regional/remote residents for categories of the demographic and clinical variables. The comparisons are reported as prevalence ratios with 95% confidence intervals. The prevalence of men living in outer regional/remote areas was elevated among older persons, those from the middle to lower end of the SEIFA distribution, those with moderate to high PSA levels (10.01–20 ng/mL) at diagnosis, those with high-risk disease, those diagnosed in public facilities, and those treated in public facilities. These elevations in prevalence persisted after adjustment for age. Conversely, adjustment for age eliminated the elevations in prevalence of outer regional/remote residence among those with higher Gleason scores. To further understand regional disparities in clinical features of disease, including diagnostic PSA, Gleason score and NCCN risk group, further analyses were performed adjusting for age at diagnosis, SEIFA, method of diagnosis and type of diagnostic facility. The adjustment for SEIFA was chiefly responsible for the change in coefficient estimates. The elevated prevalence of men living in outer regional/remote areas with high-risk disease persisted however, the disparities in prevalence of outer regional/remote residence among those with moderate to high PSA levels and low-risk disease were eliminated (Table 2). Interestingly, the prevalence ratios for higher Gleason scores (≥ 8) were once again elevated after further adjustment, though not with statistical significance.

**Table 2:** Prevalence of demographic and clinical characteristics in men with prostate cancer living in outer regional/remote areas of Tasmania relative to their counterparts living in inner regional areas.

	Unadjusted PR (95% CI)	Adjusted* PR (95% CI)	Adjusted^†^ PR (95% CI)
**Age at diagnosis**			
< 60	n.a		
60–64	**0.69 (0.55–0.86)**		
65–69	**1.26 (1.07–1.50)**		
≥ 70	**1.14 (1.02–1.28)**		
**SEIFA quintile**			
1 (Most disadvantaged)	n.a	n.a	
2	**3.12 (2.63–3.70)**	**3.13 (2.63–3.72)**	
3	**2.53 (2.03–3.14)**	**2.56 (2.04–3.20)**	
4	**0.55 (0.45–0.68)**	**0.56 (0.45–0.69)**	
5 (Most advantaged)	**0.11 (0.07–0.16)**	**0.10 (0.07–0.15)**	
**Method of diagnosis**			
TRUS	n.a	n.a	
TURP	1.13 (0.85–1.50)	0.94 (0.74–1.19)	
TP Biopsy	1.05 (0.97–1.13)	1.07 (0.99–1.15)	
Other	0.97 (0.47–1.98)	0.81 (0.40–1.64)	
**Diagnostic PSA (ng/mL)**			
≤ 4	n.a	n.a	n.a
4.01–10	0.92 (0.83–1.01)	0.99 (0.90–1.08)	1.00 (0.90–1.11)
10.01–20	**1.35 (1.13–1.62)**	**1.24 (1.02–1.49)**	1.11 (0.93–1.32)
> 20.01	1.10 (0.84–1.45)	0.91 (0.73–1.14)	0.88 (0.75–1.02)
**Gleason score**			
< 7	n.a	n.a	n.a
7 (3 + 4)	0.99 (0.84–1.17)	1.04 (0.87–1.23)	1.08 (0.90–1.3)
7 (4 + 3)	**1.27 (1.01–1.59)**	1.20 (0.94–1.53)	1.02 (0.78–1.3)
≥ 8	**1.21 (1.02–1.43)**	0.98 (0.82–1.16)	1.20 (0.99–1.46)
**NCCN risk**			
Low	**0.70** (**0.58–0.84)**	**0.75** (**0.62–0.89)**	0.88 (0.73–1.08)
Intermediate	n.a	n.a	n.a
high	**1.30** (**1.11–1.52)**	**1.31** (**1.13–1.52)**	**1.22 (1.01–1.47)**
Very high/metastatic	0.99 (0.74–1.32)	0.99 (0.74–1.31)	0.87 (0.58–1.29)
**Diagnostic facility**			
Public	n.a	n.a	
Private	**0.82 (0.76–0.88)**	**0.82 (0.76–0.87)**	
**Treatment facility (active treatment)**			
Public	n.a	n.a	
Private	**0.83 (0.72–0.95)**	**0.84 (0.73–0.97)**	

PR = Prevalence Ratio; CI = Confidence Interval; n.a. = Not Applicable; SEIFA = Socio-Economic Index of Advantage and Disadvantage; TRUS = Transrectal Ultrasound; TURP = Transurethral Resection of the Prostate; TP Biopsy = Transperineal Biopsy; PSA = Prostate Specific Antigen; NCCN = National Comprehensive Cancer Network.

*Adjusted for age at diagnosis.

^†^Adjusted for age at diagnosis, SEIFA index, method of diagnosis and type of diagnostic facility.

Table 3 shows the frequencies of types of primary treatments undertaken by individuals with PrCa living in inner regional and outer regional/remote areas, stratified by NCCN risk to restrict comparison to those with a similar disease status at diagnosis. Men from both area classifications showed similar distributions of primary treatment type across each risk group. Both inner regional and outer regional/remote individuals were equally as likely to undergo active treatment, and the specific active treatment approaches were consistent between the groups. Those belonging to the outer regional/remote cohort travelled a significantly greater distance to access treatment facilities, compared with those residing in inner regional areas.

**Table 3:** Treatment approaches and distance to treatment, stratified by NCCN risk, of men with prostate cancer in inner and outer regional/remote areas of Tasmania.

	Low-Risk		Intermediate-Risk		High-Risk		Very High-Risk/Metastatic	
	Inner regional, % (n*)	Outer regional/remote, % (n*)	Inner regional, % (n*)	Outer regional/remote, % (n*)	Inner regional, % (n*)	Outer regional/remote, % (n*)	Inner regional, % (n*)	Outer regional/remote, % (n*)
**WW/AS**	70.6% (191)	76.3% (82)	20.5% (75)	19.3% (42)	16.5% (38)	16.8% (29)	n.a. (< 5)	n.a. (< 5)
**Active**	25.8% (70)	18.8% (20)	75.5% (277)	74.5% (161)	67.8% (158)	65.9% (112)	86.5% (87)	85.0%% (48)
ADT	n.a. (< 5)	n.a. (< 5)	8.5% (31)	6.8% (15)	31.1% (72)	30.8% (53)	70.6% (71)	69.1% (39)
BT	3.1% (8)	4.3% (5)	1.6% (6)	n.a. (< 5)	n.a. (< 5)	n.a. (< 5)	n.a. (< 5)	n.a. (< 5)
RP	19.1% (52)	13.3% (14)	47.4% (174)	49.9% (108)	26.7% (62)	21.0% (36)	9.4% (9)	9.8% (6)
RT	2.5% (7)	n.a. (< 5)	17.6% (65)	16.4% (35)	9.9% (23)	13.5% (23)	4.7% (5)	n.a. (< 5)
Other	n.a. (< 5)	n.a. (< 5)	n.a. (< 5)	n.a. (< 5)	n.a. (< 5)	n.a. (< 5)	n.a. (< 5)	n.a. (< 5)
**Missing**	3.6% (10)	4.9% (5)	4.0% (15)	6.2% (13)	15.7% (36)	17.3% (30)	11.5% (12)	11.4% (6)
**Total**	100% (271)	100% (107)	100% (367)	100% (216)	100% (233)	100% (170)	100% (89)	100% (50)
**Distance (kms) to primary active treatment (median (IQR))**	10.6 (6.7–19.3)	59.7 (52.3–104.6)	10.4 (6.7–36.3)	104.5 (53.7–139)	9.7 (2.2–20.2)	104.6 (42.46–128.3)	10.4 (2.2–17.4)	108.5 (61.6–139)

NCCN = National Comprehensive Cancer Network; WW/AS = Watchful Waiting/Active Surveillance; n.a. = Not Applicable; ADT = Androgen Deprivation Therapy; BT = Brachytherapy; RP = Radical Prostatectomy; RT = Radiotherapy; kms = kilometers; IQR = Interquartile Range.

*All analyses are weighted for remoteness classification (see “Methods”). Subject numbers are rounded to the nearest integer value.

The time in days between diagnosis and commencement of any primary intervention (including WW/AS) was significantly different between the inner regional and outer regional/remote groups, as shown in Table 4. When just active treatment was considered, the difference was significant only when adjusted for age, with the relatively older individuals from outer regional/remote areas taking approximately nine days longer to commence treatment. When stratified by NCCN risk group, while men living in outer regional/remote areas with low- and high-risk disease took over 10 days longer to commence primary treatment than inner regional men, these differences were not statistically significant (Table 4).

**Table 4:** Differences in time and distance travelled to primary treatment for men with prostate cancer in inner and outer regional/remote areas of Tasmania.

	Time in days, median (IQR)	Additional time for individuals living in outer regional/remote areas	
		Unadjusted PR (95% CI)	Adjusted* PR (95% CI)
**Time to primary treatment (days)**			
Inner regional	61 (31–101)	n.a	n.a
Outer regional/remote	69 (36–116)	**9.51 (2.35–16.68)**	**11.02 (3.92–18.12)**
% of total dataset, % (n^†^)	80.1% (1222)		
**Time to primary active treatment (days)**			
Inner regional	75 (49–115)	n.a	n.a
Outer regional/remote	82 (50–131)	6.66 (− 1.05–14.38)	**9.25 (1.72–16.79)**
% of total dataset, % (n^†^)	60.1% (917)		
**Low-risk**			
Inner regional	93 (63–123)	n.a	n.a
Outer regional/remote	96 (68–185)	12.98 (− 19.05–45.01)	16.21 (− 14.47–46.89)
% of Total dataset, % (n^†^)	4.6% (70)		
**Intermediate-risk**			
Inner regional	85 (61–126)	n.a	n.a
Outer regional/remote	95 (63–133)	5.16 (− 4.17–14.49)	5.61 (− 3.72–14.93)
% of Total dataset, % (n^†^)	28.6% (437)		
**High-risk**			
Inner regional	64 (42–100)	n.a	n.a
Outer regional/remote	75 (43–136)	13.14 (− 1.66–27.93)	14.58 (− 0.50–29.65)
% of Total dataset, % (n^†^)	17.4% (265)		
**Very high-risk/metastatic**			
Inner regional	36 (16–59)	n.a	n.a
Outer regional/remote	49 (23–72)	2.78 (− 12.58–18.12)	2.97 (− 12.47–18.40)
% of Total dataset, % (n^†^)	8.1% (123)		

IQR = Interquartile Range; PR = Prevalence Ratio; CI = Confidence Interval; n.a. = Not Applicable.

*Adjusted for age at diagnosis.

^†^All analyses are weighted for remoteness classification (see “Methods”). Subject numbers are rounded to the nearest integer value.

## Discussion

Our study indicated that men from outer regional/remote areas were less prevalent amongst patients diagnosed with low-risk disease and more prevalent amongst patients diagnosed with higher PSA levels (10.1–20 ng/mL), with higher Gleason scores (≥ 8) and with high-risk disease, corroborating previous studies in other states of Australia^4^ and internationally^8–11^. Interestingly, when SEIFA was included as a covariate in these analyses, only the latter result remained significant. While the prevalence of men from outer regional/remote areas with higher Gleason scores was still close to significant, the effect of SEIFA on the prevalence of these men with low-risk disease and moderate-to-high diagnostic PSA levels was more apparent. This is not surprising given that PrCa patients from outer regional/remote areas were significantly more likely to reside in areas with lower rates of socio-economic advantage, which may inhibit their access to healthcare, including regular screening check-ups. Another possible factor contributing to the higher rates of advanced disease diagnosed amongst this cohort could be the higher PSA monitoring rates seen in metropolitan regions of Australia and New Zealand compared to rural regions^2,7,8^. As a rise in PSA is one of the few strategies for early detection of PrCa, limited access to this testing could result in the delay of diagnosis until the disease becomes symptomatic.

The established pattern of advanced disease, in addition to poorer survival outcomes, in Australian PrCa patients outside of urban regions has long been observed^2,4,12–14^, yet the reasons for this remain unclear. Registries such as PCOR-TAS provide valuable insight into demographic and clinical variables that may influence these factors. It has previously been suggested that discrepancies in diagnostic clinical features and treatment practices between inner regional and outer regional/remote cohorts could be explained by a combination of clinical, patient-related, accessibility, and healthcare-related factors^15^. Likely due to the small size and relatively low population density of Tasmania, the majority of healthcare services are located in urban regions. All diagnostic and treatment facilities accessed by the participants of this study are located within inner regional postcodes, potentially presenting a serious accessibility barrier to those of outer regional/remote habitations, especially as these areas tend to be of lower socio-economic advantage. The required travel time, the cost of transport, access to, and the timely nature of that transport all could play a real and negating role in obstructing men in the outer regional/remote cohort from accessing healthcare in a timely and regular fashion. This may not only complicate an individual’s means of seeing a clinician for a specific concern, but also diminishes their likelihood of seeking regular check-ups. It has been shown that the personal and time costs of travel required of regional and remote residents to access urban medical specialist services are significant, costs that can be substantially reduced by access to local or visiting specialist services^16^.

In contrast to previous studies^2,3,15,17^, no significant difference in primary treatment type was found between the two cohorts. This remained consistent when patients were stratified by risk group, where both inner regional and outer regional/remote cohorts were as likely to undergo active treatment. However, while not significant, a greater proportion of low-risk outer regional/remote patients underwent WW/AS (80.26% vs. 73.23%), which may have been influenced by their lack of proximity to treatment facilities. As expected, active treatment increased as disease risk increased, with consistent treatment modalities seen in both cohorts. Radical prostatectomy was by far the most common treatment approach for individuals diagnosed with intermediate-risk disease but as disease risk increased, a wider range of active therapies were undertaken, with ADT, in particular, becoming progressively more common. Previously, it has been shown that residents of outer regional/remote areas were significantly less likely to undergo radical prostatectomy compared with residents of capital cities^2,3,17^. In fact, a reduced level of surgical intervention is observed for many non-life-threatening conditions in Australian outer regional/remote residents compared to metropolitan residents^15^.

It could be hypothesised that the relatively small number of centrally located healthcare facilities in Tasmania, characteristic of less densely populated regions, have brought about equity of treatment, as patients from all areas are accessing the same clinicians and facilities. In fact, facilities for surgery and radiation oncology are limited to two and three inner regional areas around the state, respectively. There was, however, a notable time delay between the date of diagnosis and the commencement of primary active treatment in men from outer regional/remote areas, although this was only significant when adjusted for age at diagnosis. Furthermore, and potentially impacting treatment commencement times, patients from inner regional and outer regional/remote cohorts were differentially dispersed between private and public facilities. Inner regional patients were significantly more likely to be both diagnosed and treated in a private healthcare setting. Given that many of the private facilities and their public counterparts are located in the same postcode, these disparities may be a reflection of the significantly different socio-economic status of the groups.

This study is the first to utilise the wholly regional population-based cohort registry, PCOR-TAS. This registry comprises a wealth of demographic and clinical variables allowing the evaluation of clinical practices in regional PrCa patients, and the use of state-wide clinical registry data is a powerful asset. The ability to map detailed diagnostic and treatment data across the state allows for the observation and interpretation of current patient and clinical patterns and trends. As a relatively new registry, some limitations must be acknowledged. The dataset presented here only accounts for approximately 65% of all Tasmanian PrCa patients diagnosed over this study period. There are a number of reasons for this discrepancy, including participants who opt out, cases who were diagnosed interstate, cases diagnosed on coroner’s report, etc. However, the main reasons for this discrepancy are urologists not participating in the registry (n = 1, accounts for 5–10% of cases) or urologists who were seeing patients before being recruited to PCOR-TAS (accounts for 15–20% of cases over the study period). Missing TNM data in a large proportion of patients (~ 48%) may also have led to some being misclassified with regards to NCCN risk, especially for the Very High/Metastatic category that relies solely on TNM data. Another limitation is the lack of comorbidity data, preventing us from determining whether disparities in disease severity and commencement of treatment are affected by differences in general health between the two groups. Finally, given the high five-year survival rate for PrCa, the registry is too “young” to have sufficient recurrence and survival data for outcome analyses.

Though currently limited in size and duration, with some incomplete data, increasing participants and the collection of outcomes data (e.g., survival data), will ensure that our ability to monitor PrCa trends in a regional setting will also continue to evolve and improve. This will consequently provide more informed and precise recommendations to clinicians, healthcare providers, and policymakers, resulting in more equitable access to healthcare facilities and better outcomes for men living in regional and remote areas across the world.

## Methods

### Prostate Cancer Outcomes Registry-Tasmania (PCOR-TAS)

PCOR-TAS was established in 2015, with an aim to improve all aspects of the quality of care for men diagnosed with PrCa. The opt-out registry is an ongoing initiative that records data on the diagnosis, treatment, outcomes, and quality of life for all Tasmanians diagnosed with PrCa^18^. Details of the registry, including data collection methods, have been described previously^19^. As of the 19th of July 2019, 1,526 men had been recruited into PCOR-TAS, with ~ 3% having opted out of the registry. PCOR-TAS participants consented to their data being used for ethically approved research. Ethics approval for this study was obtained from the Tasmanian Health and Medical Human Research Ethics Committee, Australia (project ID: H0017095) and was performed in accordance with the principles of the Declaration of Helsinki.

### Data extraction

De-identified, record unit level data were extracted from PCOR-TAS for all Tasmanian PrCa cases enrolled in the registry between 01/02/2015 and 19/07/2019 (N = 1526). Standard data cleaning and quality control checks were undertaken. As not all variables were available for all PCOR-TAS participants, numbers vary between each of the nominated analyses described below.

### Remoteness classification

The ABS utilises a ‘remoteness areas’ classification within the Australian Statistical Geography Standard to divide Australia into five categories of remoteness on the basis of a measure of relative access to services, measured via the Accessibility and Remoteness Index of Australia. Each Australian postcode is assigned a remoteness classification. Residential postcode information was provided by PCOR-TAS for all individuals, who were subsequently divided into two groups dependent on whether they lived in an inner regional area (‘Inner Regional’), or an outer regional, remote, or very remote area (‘Outer Regional/Remote’).

Because some postcodes comprised both inner regional and outer regional/remote areas (n = 23; 22.5%), the subjects from those postcodes (n = 359; 23.5%) were duplicated in the dataset. They were categorised in the first instance as inner regional and assigned a weight equal to the proportion of the postcode classified as inner regional, and in the second instance as outer regional/remote and assigned a weight equal to the proportion of the postcode classified as outer regional/remote. All other subjects were included only once, categorised according to the remoteness status of their postcode and assigned a weight of unity. All analyses were weighted, and robust standard errors were estimated to account for the lack of independence between duplicated individuals. The subject numbers reported in tables have been rounded to avoid the distraction that may have arisen had they been reported as non-integers.

### Variable categorisation

The ABS-developed SEIFA (2016) were used to group participants by relative socio-economic advantage and disadvantage. Specifically, the Index of Relative Socio-Economic Advantage and Disadvantage (IRSAD) was used to classify postcodes by deciles. The IRSAD incorporates a range of census variables to rank areas on a continuum, from most disadvantaged (1^st^ decile) to most advantaged (10th decile). For analyses, these categories were grouped as fifths.

Age at diagnosis was divided into five-year increments from age 50 to age 69 years, with those outside that range grouped as < 50 years or ≥ 70 years respectively. Method of diagnosis was categorised as: (1) Transrectal Ultrasound (TRUS), (2) Transurethral Resection of the Prostate (TURP), (3) Transperineal Biopsy (TP), and (4) Other. PSA level at diagnosis was grouped into four categories: (1) < 4 ng/mL, (2) 4.01–10 ng/mL, (3) 10.01–20 ng/mL, and (4) > 20 ng/mL. Gleason score at diagnosis was separated into (1) score < 7, (2) score = 7 (3 + 4), (3) score = 7 (4 + 3), and (4) score ≥ 8. The National Comprehensive Cancer Network (NCCN) have established risk criteria for disease progression that are used to classify PrCa patients into low-, intermediate-, high-, and very high-risk/metastatic disease categories (Supplementary Table S1)^20^. This system utilises clinical TNM scores (Tumour, Node, and Metastasis staging), as well as PSA levels and diagnostic Gleason score to predict an individual’s risk of disease progression. Here, participants were stratified into these risk categories based predominantly on PSA level and Gleason score as a large proportion (~ 48%) of TNM data were missing (Supplementary Table S2).

Both diagnostic and treatment facilities were classified according to their public or private status. Primary treatment recorded within 12 months of diagnosis was categorised as follows: (1) Watchful Waiting/Active Surveillance (WW/AS), (2) Chemical/Surgical Androgen Deprivation Therapy (ADT), (3) Brachytherapy (BT), (4) Radical Prostatectomy (RP), (5) Radiotherapy (RT), and (6) Other (i.e., Chemotherapy, Focal Gland Ablation Therapy). The registry did not differentiate between watchful waiting and active surveillance. Active treatment was defined as all of the above treatments other than WW/AS. Time to treatment was calculated using the number of days between date of diagnosis and the date of first recorded treatment. Distance to primary treatment facility was calculated using the participant’s residential postcode and the postcode of the treatment facility.

### Statistical analyses

The distributions of the demographic and clinical factors are reported as percentages and frequencies for participants from the inner regional and outer regional/remote areas. In accordance with our primary hypothesis, *χ*^2^ tests of independence were conducted to assess differences in the distributions.

Regression analyses were conducted with the demographic and clinical factors as outcome variables and the inner regional or outer regional/remote classification as the explanatory factor. The methods of analyses included linear regression for continuous variables, log binomial regression for binary variables, and log multinomial regression for categorical variables with more than two attributes^21^. The results of the log binomial and log multinomial regression analyses are reported as prevalence ratios with 95% confidence intervals. The prevalence ratios compare the probability of a fitter outcome for residents of outer regional/remote areas to the probability of the same outcome for residents of inner regional areas. Multivariable analyses, initially adjusting for age at diagnosis, and further adjusting for age at diagnosis, SEIFA index, method of diagnosis, and type of diagnostic institute, were performed. For linear regression, a transformation of the outcome variable was undertaken prior to analysis of the skewed data on age and distance or time to treatment. In those cases, the results are reported on the natural scale after “back-transformation”. The summary data in Table 4 are reported as medians and interquartile ranges. All analyses were performed using Stata/SE 16.1 (StataCorp, TX, USA).

## Supplementary Information

Supplementary Information.
