# Supplementary material for: Urban–rural prostate cancer disparities in a regional state of Australia
Source: Sci Rep. 2022 Feb 22;12:3022. doi: 10.1038/s41598-022-06958-2 (PMC8863814; doi:10.1038/s41598-022-06958-2)
Supplement: Supplementary file 1 — Supplementary Information. [file 41598_2022_6958_MOESM1_ESM.docx]

**Supplementary Table One** – NCCN Risk Grouping Criteria

| Risk Group | Criteria |
| --- | --- |
| Low | Clinical T1-T2a stage AND GS <6 AND PSA <10 ng/mL |
| Intermediate | Clinical T2b-T2c stage OR GS =7 OR PSA 10-20 ng/mL |
| High | Clinical T3a stage OR GS ≥8 OR PSA >20 ng/mL |
| Very High/Metastatic | Clinical T3b-T4, any T, any N, M1 |

NCCN = National Comprehensive Cancer Network; GS = Gleason Score; PSA = Prostate Specific Antigen. Where TNM data were missing, NCCN risk grouping was based on GS and PSA data.

**Supplementary Table Two** – TNM data used for NCCN risk grouping

| TNM Score | % (n*) | |
| --- | --- | --- |
| Clinical T1-T2a stage | 30.9% | (472) |
| Clinical T2b-T2c stage | 7.6% | (116) |
| Clinical T3a stage | 3.2% | (50) |
| Clinical T3b-T4, any T, any N, M1 | 10.3% | (157) |
| Missing | 47.9% | (731) |

TNM = Tumour, node, and metastasis staging; NCCN = National Comprehensive Cancer Network; *All analyses are weighted for remoteness classification (see methods). Subject numbers are rounded to the nearest integer value.
